# Associations among phthalate exposure, DNA methylation of *TSLP*, and childhood allergy

**DOI:** 10.1186/s13148-021-01061-1

**Published:** 2021-04-09

**Authors:** Wan-Ru Wang, Nai-Tzu Chen, Nai-Yun Hsu, I-Ying Kuo, Hsin-Wen Chang, Jiu-Yao Wang, Huey-Jen Su

**Affiliations:** 1 Department of Environmental and Occupational Health, College of Medicine, National Cheng Kung University, Cheng-Hsing Campus, No. 1, University Road, Tainan City, Taiwan; 2 Research Center of Environmental Trace Toxic Substances, National Cheng Kung University, Tainan, Taiwan; 3 Department of Pharmacology, College of Medicine, National Cheng Kung University, Tainan, Taiwan; 4 Department of Pediatrics, College of Medicine, National Cheng Kung University, Tainan, Taiwan

**Keywords:** Phthalate, DNA methylation, TSLP, Allergic disease

## Abstract

**Background:**

Dysregulation of thymic stromal lymphopoietin (TSLP) expressions is linked to asthma and allergic disease. Exposure to phthalate esters, a widely used plasticizer, is associated with respiratory and allergic morbidity. Dibutyl phthalate (DBP) causes TSLP upregulation in the skin. In addition, phthalate exposure is associated with changes in environmentally induced DNA methylation, which might cause phenotypic heterogeneity. This study examined the DNA methylation of the *TSLP* gene to determine the potential mechanism between phthalate exposure and allergic diseases.

**Results:**

Among all evaluated, only benzyl butyl phthalate (BBzP) in the settled dusts were negatively correlated with the methylation levels of *TSLP* and positively associated with children’s respiratory symptoms. The results revealed that every unit increase in BBzP concentration in the settled dust was associated with a 1.75% decrease in the methylation level on upstream 775 bp from the transcription start site (TSS) of *TSLP* (*β* =  − 1.75, *p* = 0.015) after adjustment for child’s sex, age, BMI, parents’ smoking status, allergic history, and education levels, PM_2.5_, formaldehyde, temperature; and relative humidity. Moreover, every percentage increase in the methylation level was associated with a 20% decrease in the risk of morning respiratory symptoms in the children (OR 0.80, 95% CI 0.65–0.99).

**Conclusions:**

Exposure to BBzP in settled dust might increase children’s respiratory symptoms in the morning through decreasing *TSLP* methylation. Therefore, the exposure to BBzP should be reduced especially for the children already having allergic diseases.

**Supplementary Information:**

The online version contains supplementary material available at 10.1186/s13148-021-01061-1.

## Background

An allergy is a complex and multifactorial condition characterized by the hypersensitivity of the immune system to any substance in the environment, such as dust mites, pollen, and fungal spores. The potential mechanisms of allergies are not yet completely understood, but genetics and biological pollutants are major risk factors for the development and exacerbation of allergic diseases [1–4]. In addition, exposure to chemicals in indoor environments is associated with allergic sensitization of the respiratory tract, manifesting in conditions such as rhinitis and asthma [5–9]. The overall increase in the variety and use of chemical pollutants since the industrial era, especially phthalates that are released from soft polyvinyl chloride materials, is a critical risk factor for the prevalence of allergic diseases in modern society [10–13].

Phthalates are widely used as stabilizers and plasticizers in common consumer products [14], such as raincoats, footwear, food packaging, personal care products, medical equipment, toys, insecticides, and building materials [15]. The annual global production of phthalates is estimated to be 8.4 million tons [16, 17]. Phthalates are ubiquitous in indoor air, dust, water, and food owing to their weak intermolecular forces [18]; therefore, people are commonly exposed to phthalates through diet, inhalation, and dermal contact [12, 13, 19, 20]. Xu et al. (2009) built a model to predict di-2-ethylhexyl phthalate (DEHP) emissions and potential exposures and demonstrated that phthalate exposure in childhood was higher than that in adults from not only food ingestion but also inhalation and dermal contact [21]. In effect, a study used the children sampled for the German Environmental Survey on children (GerES IV) (*n* = 254) and a non-occupationally exposed German population for adults (*n* = 85) [22, 23]. The studies of Koch (2006) [24] evaluated the urinary phthalate concentrations of children aged 3–14 years, German population aged 7–63 years and compared the exposure level with reference dose (RfD). They detected the phthalate metabolites in the urine and observed that DEHP intake was higher in children than in adults. The median of DEHP daily intake is 7.7 μg/kg body weight/day for children and 5.6 μg/kg body weight/day for adults. Among those 254 children analyzed, there was 10% (26/254 * 100%) of children assessed exceeded the RfD of the US EPA, and the DEHP exposure in some children was even up to 20 times higher than RfD [24]. An exposure assessment also resulted in higher internal exposure values via human biomonitoring data than ambient concentration, especially in children [25]. Moreover, phthalate exposure may adversely affect childhood development [26–34]. Indoor phthalate exposure has been shown to influence the pathogenesis of allergic diseases [10–13]. Additionally, a dose–response relationship was observed between phthalates in settled dust and wheezing in preschool children [10, 35, 36]. Instead of acting as allergens, phthalates promote and aggravate allergic diseases by functioning as an adjuvant to disrupt the immune system [37–42]. Studies have reported that exposure to phthalates can influence gene expression and cell function. A Taiwanese study reported that the higher mono-(2-ethyl-5-hydroxyhexyl) phthalate (MEHHP) concentration found in superoxide dismutase 2 TT genotypes was more correlated to asthma than was CC types and it suggested that genetic variants might modify the association between phthalate exposure and asthma [43].

Gene function and expression are modulated by genetic and epigenetic factors. The term epigenetics describes the complex gene–environmental interactions that are associated with disease development and cell differentiation [44–46]. DNA methylation is an epigenetic mechanism in which methyl groups are added to the cytosine of cytosine–phosphate–guanine sites (CpG sites), which alter the activity of the DNA segment without changing the DNA sequence. Moreover, DNA methylation is a product of gene-environment interactions, which provides a stable and reversible reaction of gene silence [47, 48]. Changes in DNA methylation levels mediate the associations of environmental exposures with asthma and allergy [49–52]. Some cross-sectional studies using the Isle of Wight birth cohort have concluded that *leptin* (*LEP)* and *interleukin-4 Receptor* (*IL4R)* methylation are both negatively correlated with asthma and that the interaction between DNA methylation and single nucleotide polymorphism (SNP) might influence lung function [53–56]. Numerous reports have indicated that early life phthalate exposure may modify DNA methylation, thereby mediating health outcomes [50, 51, 57–62]. The Childhood Environment and Allergic Diseases Study from Taiwan suggested that higher urine phthalate levels were related to lower *tumor necrosis factor-α (TNF-α)* methylation levels, potentially exacerbating asthma [51].

The immune system plays a vital role in the worsening of pathologic allergic diseases. Depending on the innate immune response–activating capacity, dendritic cells and naïve T cells can differentiate into T-helper (T_H_1-, T_H_2-, T_H_9-, T_H_17-, or T_H_22-type) memory and effector cells [63–65]. During an allergic response, naïve T cell activation induces the expression of T_H_1 and T_H_2 subtypes, leading to the production of inflammatory cytokines. The shift in T_H_1/T_H_2 homeostasis toward the T_H_2 phenotype contributes to the inflammation of allergic diseases and related symptoms [66]. Thymic stromal lymphopoietin (TSLP) is crucial for the maturation of antigen-presenting cells (APCs) and for skewing the T-helper immune response toward the T_H_2 phenotype, which is typical of allergic inflammation; TSLP can be released on allergen stimulation [67, 68]. TSLP is an interleukin (IL)-7–like cytokine that enhances a T_H_2 cell-polarizing signal and OX40 ligand, which provide a microenvironment to trigger dendritic cell–mediated T_H_2 inflammatory responses [67, 68]. Thus, TSLP is essential in inducing the differentiation of naïve T cells into T_H_2 cells in the pathogenesis of allergic diseases. Furthermore, in an animal study, contact hypersensitivity was inducted using hapten fluorescein isothiocyanate (FITC), demonstrating that exposure to FITC, in combination with dibutyl phthalate (DBP) compared with FTIC alone, can upregulate the TSLP protein, suggesting that the TSLP protein is an endogenous mediator of the adjuvant effect of DBP [69]. However, whether a phthalate exposure–induced imbalance of the T_H_1–T_H_2 pathway is associated with the regulation of DNA methylation is unclear. Thus, this study investigated the relationship of phthalates exposure, including phthalates in the settled dusts and phthalate metabolites in the urine, with changes in methylation levels of TSS on *TSLP* promoter sites. Based on the results, the present study also attempted to clarify the underlying effects between phthalate exposure and childhood allergic diseases with regard to epigenetics.

## Results

The study participants were 7.05 ± 1.19 years old and had an average body mass index of 16.26 ± 2.08; 56 participants were boys, and 34 participants were girls. The parents of most participants (87.8%) did not smoke. More than half of the participants were diagnosed as having allergic diseases by pediatricians, and nearly 41% of them experienced respiratory symptoms on waking up (Table 1).

**Table 1:** Descriptive characteristics of subjects

	*N*	%
Age (Mean ± SD)	7.05 ± 1.19	
BMI (Mean ± SD)	16.26 ± 2.08	
*Sex*		
Female	34	37.8
Male	56	62.2
With breastfeed	52	57.8
*Parental smoking status*		
Yes	11	12.2
No	79	87.8
*Parental education level higher than college*		
Mother	49	44.4
Father	52	57.8
*Parental income higher than 40,000 NT dollars*		
Mother	17	18.9
Father	50	55.6
*Family history of allergic disease*		
Mother	26	28.9
Father	29	32.2
*Physician‐diagnosed allergic disease*		
Yes	52	57.8
No	38	42.2
*Parents‐reported symptoms during the week of household investigation*		
Respiratory symptoms		
During sleeping	16	17.8
While getting up	37	41.1
During daytime	25	27.8
Eye‐related symptoms	24	26.7

Table 2 presents the profiles of phthalate exposure and DNA methylation. Among the parental compounds of phthalates in settled dusts, DEHP had the highest concentration, followed by DBP and benzyl butyl phthalate (BBzP). Among urine metabolites, monobutyl phthalate (MBP) had the highest concentration, followed by MEHHP and mono-(2-ethyl-5-oxohexyl) phthalate (MEOHP). Regarding the median serum levels of the eight cytokines measured, the levels of inflammatory-related cytokines (interleukin-6 (IL-6), interleukin-8 (IL-8), and TNF-α) were the highest, followed by those of T_H_2 pathway-related cytokines (interleukin-4 (IL-4), interleukin-5 (IL-5), and interleukin-13 (IL-13)) and T_H_1 pathway-related cytokines (interleukin-12 (IL-12) and Interferon-γ (IFN-γ)). The median *TSLP* methylation percentage was 17%.

**Table 2:** Concentration of PAEs, DNA methylation, and RNA levels

	*N* (total)	Positive detection*	
		*N*^Δ^	Median (range)
*Parental compounds of phthalates (μg/g dust)*^#^			
DMP	87	11	2.43 (1.98–18.87)
DEP	87	16	3.08 (2.12–8.93)
DBP	78	78	18.61 (5.26–159.75)
BBzP	84	55	6.11 (1.94–48.53)
DEHP	85	85	909.03 (78.98–2769.2)
*Metabolism of phthalates (μg/g Cr)*^¥^			
MMP	90	90	6.64 (1.11–188.64)
MEP	90	90	15.60 (2.29–540.08)
MBP	90	90	52.76 (17.28–445.56)
MBzP	90	90	5.31 (0.97–217.16)
MEHP	90	90	10.11 (1.79–361.44)
MEHHP	90	90	45.12 (10.17–2276.0)
MEOHP	90	90	42.62 (10.21–1769.9)
*Cytokine (pg/ml)*^†^			
IL-4	90	11	1.77 (1.14–12.44)
IL-5	90	47	1.51 (1.03–3.88)
IL-6	90	20	1.78 (1.15–9.78)
IL-8	90	81	2.12 (1.10–7.90)
IL-12	90	12	1.51 (1.11–8.52)
IL-13	90	16	1.92 (1.16–11.09)
IFN-r	90	44	1.56 (1.02–24.99)
TNF-α	90	39	1.72 (1.12–4.01)
*DNA methylation percentage of TSLP promoter (%)*			
Position − 775	90	90	17 (7–27)
*mRNA expression level*^¶^			
*TSLP*	90	18	9.71 (0.08–1931.49)

^*^Samples with levels within the concentration range of calibration curve

^Δ^The number of samples with quantifiable concentrations (i.e., within the calibration range)

^#^The parental compounds of phthalates were analysis from settle dust samples

^¥^The metabolism of phthalates were analysis from urine samples

^†^Cytokine were analysis from serum samples

^¶^Percent of reference/housekeeping genes (Actin Beta (ACTB))

The *TSLP* methylation level was associated with messenger RNA (mRNA) levels (Additional file 1: Figure S1, *r* =  − 0.553, *p* = 0.01).

**Fig. 1:**
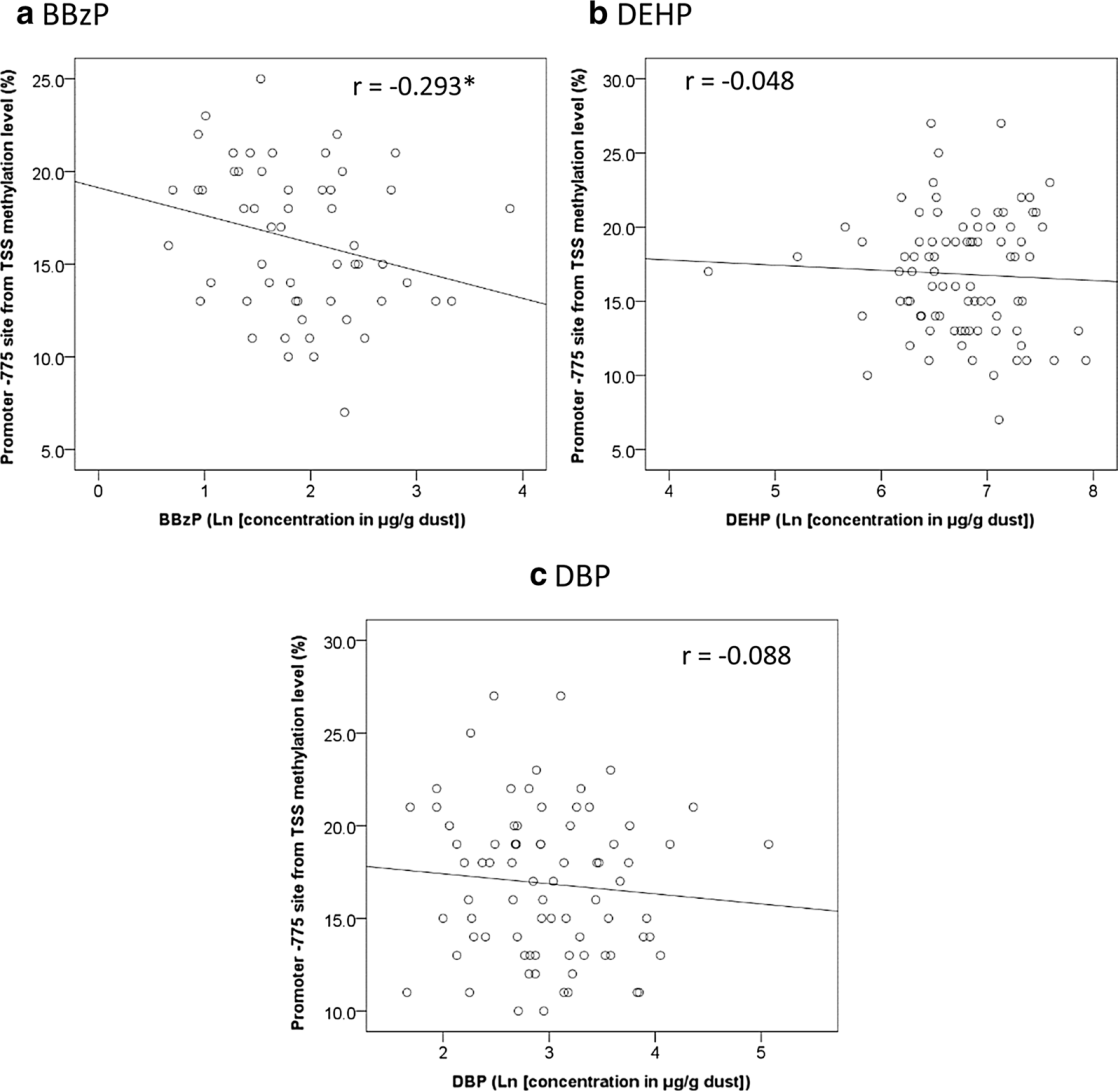
The correlation between parental compounds of phthalate in the settle dust and *TSLP* DNA methylation level in buffy coat. *p < 0.05, by Spearman correlation coefficient

**Fig. 2:**
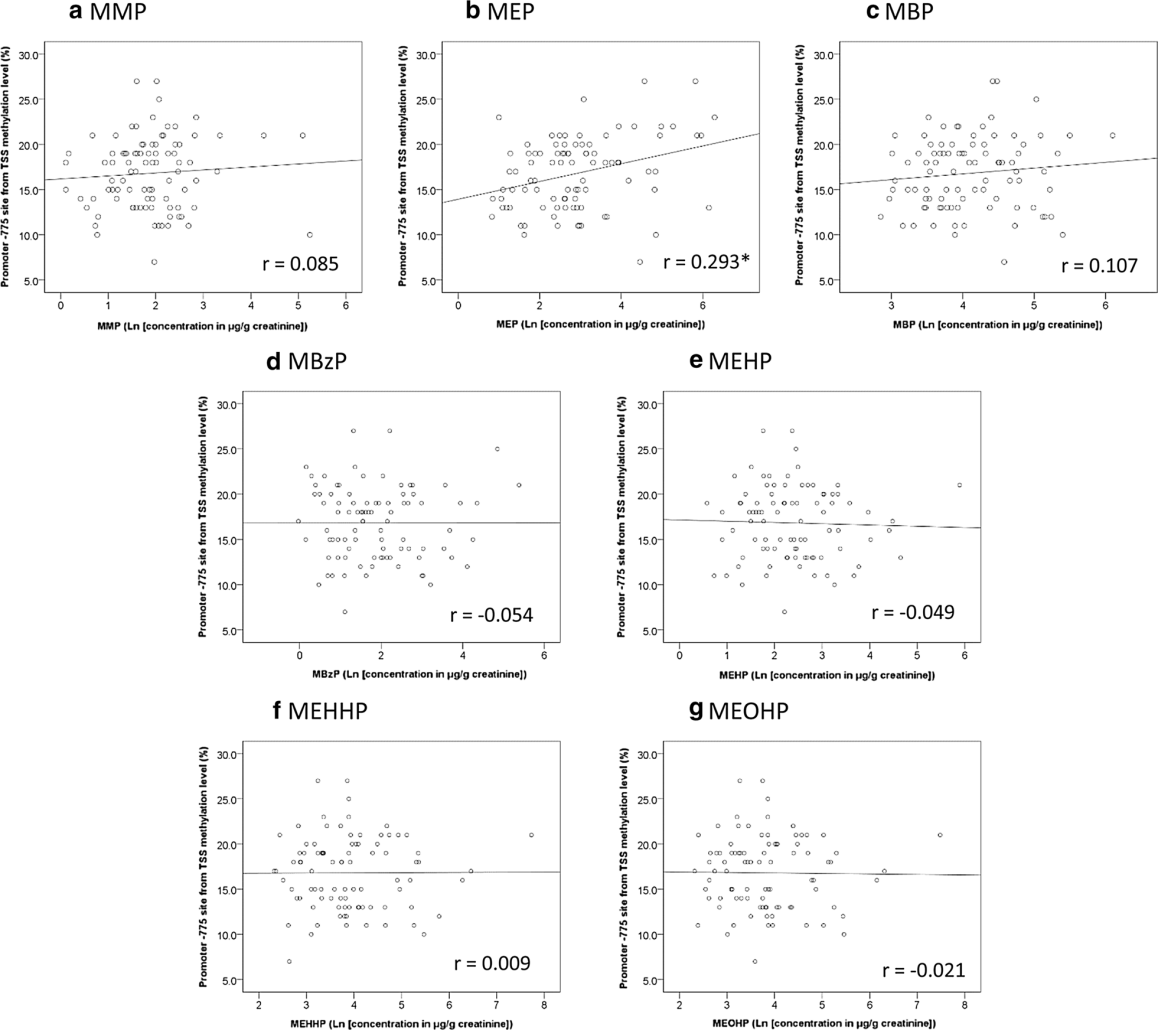
The correlation between phthalate metabolites in the urine and *TSLP* methylation levels in buffy coat. *p < 0.05, by Spearman correlation coefficient

Figure 1 shows the correlation between phthalate exposure in settle dust and the methylation levels of the upstream 775 bp from the TSS on *TSLP* promoter sites in buffy coat. The DNA methylation level was significantly and negatively correlated with BBzP concentration (Fig. 1a, *r* =  − 0.293, *p* = 0.03) but not significantly correlation with DEHP and DBP (Fig. 1b, c, *p* > 0.05 for both). The association of DNA methylation with diethyl phthalate (DEP) and dimethyl phthalate (DMP) was not analyzed because 84% and 70% samples of DEP and DMP, respectively, were unquantifiable (Table 2). The relationships between DNA methylation levels of phthalate metabolites in urine are illustrated revealed in Fig. 2. The *TSLP* methylation level was significantly associated with only monoethyl phthalate (MEP) concentration (Fig. 2b, *r* = 0.293, *p* = 0.005).

Generalized linear regression was used to evaluate the effect of BBzP and MEP on *TSLP* methylation levels with the adjustment of confounders. A significantly negative association was observed between BBzP in settled dust and *TSLP* methylation levels (Table 3).

**Table 3:** The association of Phthalate exposure and *TSLP* methylation level

	*N*	*β* (SE)^a^	95% CI
Crude model			
BBzP in settled dust^§^	55	− 1.49 (0.76)	− 2.989, − 0.004
MEP in urine^§^	90	0.98 (0.31)*	0.376, 1.59
Final model ^b^			
BBzP in settled dust^§^	55	− 1.75 (0.72)*	− 3.162, − 0.337
MEP in urine^§^	81	0.97 (0.35)*	0.274, 1.657

^a^Generalized linear model, *p < 0.05

^b^The model was adjusted for gender, age, BMI, PM_2.5_, formaldehyde, temperature and relative humidity, parents smoking status, allergic history, and education levels

**Fig. 3:**
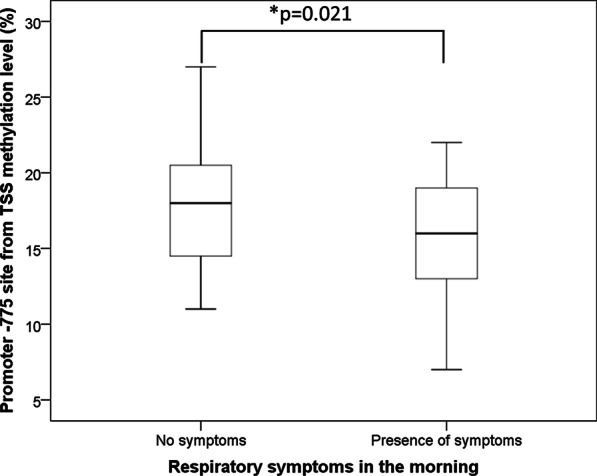
The difference between *TSLP* methylation level and presences of symptoms. *p < 0.05, by Mann–Whitney *U* test

An increase in BBzP concentration in settled dust by one unit led to a 1.75% decrease in *TSLP* methylation levels after adjustment for confounders (*β* =  − 1.75, *p* = 0.015). However, *TSLP* gene methylation was positively associated with urine MEP concentration (*β* = 0.97, *p* = 0.006). Furthermore, children with morning respiratory symptoms had significantly lower *TSLP* methylation levels than those without such symptoms (14% vs. 18%, *p* = 0.021) (Fig. 3). Logistic regression analyses indicated that a 1% increase in methylation levels was associated with a 20% decrease in the risk of morning respiratory symptoms (odds ratio (OR) = 0.80, 95% CI 0.65–0.99; Table 4).

**Table 4:** Effects of *TSLP* methylation level on respiratory symptoms

	*N*	OR^a^ (95% CI)	*p*-value
Crude model	54	0.83 (0.71–0.97)	0.021^*^
Final model^b^	52	0.80 (0.65–0.99)	0.036^*^

^a^Logistic regression, *p < 0.05

^b^The model was adjusted for gender, age, BMI, PM_2.5_, formaldehyde, temperature and relative humidity, parents smoking status, allergic history and education levels

## Discussion

Our findings indicated that higher BBzP exposure is associated with lower *TSLP* methylation levels (Fig. 1 and Table 3). Epidemiological and experimental studies have demonstrated the significant associations between phthalate concentration in indoor dust and allergic diseases [10, 12, 13, 35, 36, 70, 71]. Our previous study revealed that children with asthma had significantly higher levels of BBzP than those without asthma [12]. Moreover, phthalate exposure was found to alter the balance of T_H_1/T_H_2 cytokines to T_H_2 responses. An in vivo study indicated that phthalate in combination with FITC may trigger TSLP production and that phthalate increased both NF-κB signaling pathway activation and *TSLP* expression [43]. In our study, we observed a correlation between *TSLP* methylation levels and BBzP but not monobenzyl phthalate (MBzP). An epidemiological study revealed that the correlation between BBzP and MBzP was not strong (*r* = 0.24 and 0.21) [12, 72] and that respiratory allergic diseases were more strongly correlated with BBzP in dust than MBzP in urine [10, 12, 71]. Our results are consistent with these findings. Our findings supported that BBzP in settled dust is indicative of the phthalates that are inhaled and irritate the immune system to induce an allergic response. This study also found a positive correlation between *TSLP* methylation and MEP in urine, which was inconsistent with our assumptions that phthalate exposure may result in the hypomethylation of *TSLP* and thereby induces allergic diseases. The negative relationship between expression of *TSLP* and respiratory symptoms/allergic diseases has been established in our and previous studies [73–76]. However, the impact of MEP on allergic diseases have not been confirmed. A longitudinal study also found a negative correlation between urinary MEP and allergic rhinitis and positive correlation between urinary MEP and asthma [77]. In addition, our and the study of Callesen [78] showed no significant relationship between MEP and respiratory symptoms/allergic diseases. Therefore, we inferred that the exposure of MEP could not induce allergic symptoms through the changes in the methylation levels of *TSLP*. The positive correlation between MEP and *TSLP* methylation levels might be because another factors that associated with both of MEP and *TSLP* rather than the direct effect of MEP on *TSLP*. Further researches are needed to address the correlation between MEP and allergic disease and the potential mechanism between MEP and *TSLP* methylation.

In this study, lower *TSLP* methylation levels were found in children with morning respiratory symptoms (Fig. 3 and Table 4). Our results also revealed a negative correlation between *TSLP* methylation level and *TSLP* gene expression (Additional file 1: Figure S1), indicating that a decline in *TSLP* methylation level upregulates *TSLP* gene expression, thereby increasing the risk of morning respiratory symptoms. The results of previous studies agree with our findings [49, 79–83]. Increased *TSLP* expression, caused by decreased *TSLP* methylation level, has been linked to the occurrence and exacerbation of atopic diseases such as atopic dermatitis, asthma, and allergic rhinoconjunctivitis [49, 79–83]. Allergic diseases occur due to a complex process of allergen-specific T_H_2 cell activation by APCs followed by cytokine production [84–87]. For example, a study using an animal model of atopic dermatitis revealed that increased *TSLP* expression directly stimulated Group 2 Innate Lymphoid Cells (ILC2s), which are key regulators and effectors in type 2 immunity, and enhanced the production of interleukin-33 (IL-33) and interleukin-25 (IL-25) [68, 88, 89]. Moreover, increased *TSLP* expression may mediate immunopathology by enhancing OX-40 and TH2 signaling and causing them to produce T_H_2 subtype and inflammatory cytokines [90–93]. This mechanism is supported by our finding that *TSLP* methylation levels were negatively correlated not only with the *TSLP* expression and the occurrence of respiratory symptoms in the morning but also the production of TNF-α that is inflammatory-related cytokine (Additional file 1: Figure S3a). Moreover, a slightly positive correlation between *TSLP* mRNA expression and TNF-α concentration was observed (Additional file 1: Figure S3b).

Overall, this study demonstrated that the exposure to the higher BBzP levels in settled dusts was related to the decline in *TSLP* methylation, which could increase *TSLP* expression levels and thereby increases the risk of the presence of respiratory symptoms in the morning. Both epidemiological and experimental studies have shown the significant associations between the exposure to phthalate in indoor dusts and allergic diseases [10, 12, 35, 36, 70, 71]. However, as our best knowledge, this is the first study to indicate the underlying mechanism between phthalate exposure and childhood allergy in term of DNA methylation of *TSLP* gene, which plays an important role in the mechanism of allergic disease.

Up to now, no study focus on the functionality of this specific methylation site in the control of TSLP gene expression. We observed a significant and negative correlation between *TSLP* methylation levels and *TSLP* gene expression levels (Additional file 1: Figure S1) as well as between *TSLP* methylation levels and TSLP protein amounts (Additional file 1: Figure S2a), implying that the upstream 775 bp from the TSS of *TSLP* methylation might regulate *TSLP* expression and the occurrence of allergic symptoms (Additional file 1: Figure S2b). However, the mRNA levels were detected in only 18 samples in our study (Table 1). Tolerance for freeze–thaw events is also tissue-type dependent. Tissue storage at − 80 °C can preserve DNA and protein for many years, but RNA starts degrading at 5 years [94]. Thus, we used the Chi-square test and Mann–Whitney *U* test to assess differences in *TSLP* methylation levels and participant characteristics between 90 and 18 samples, and no significant difference was noted.

This is the first study to indicate a potential mechanism between phthalate exposure and childhood allergy in terms of *TSLP* methylation. We observed upstream gene modification between phthalate exposure and allergic symptoms, which may be used to reduce the severity of allergic symptoms by altering *TSLP* methylation. In addition, we adjusted the effects of other air pollution levels on the changes of DNA methylation in the final model.

This study has some limitations. First, although this study identified the association of *TSLP* methylation with phthalate exposure and allergic symptoms, the cross-sectional design precluded the determination of a causal relationship, which should be elucidated in future studies. Second, DNA was isolated from the buffy coat, a heterogeneous cell population that includes white blood cells and platelets. However, cells may be lysed during storage, which made us unable to consider the cell-type heterogeneity. Cell-type heterogeneity, which was correlated with age, was shown to affect DNA methylation [95]. Nevertheless, in our study, DNA methylation levels of samples collected from children aged 3–12 years were similar. Additionally, the age effect was adjusted in the final model, removing concern for cellular heterogeneity. The third limitation is DNA degradation due to long-term storage. However, because the storage period for all samples was similar, we assumed that the amount of DNA degradation in all samples was similar. Moreover, the *TSLP* methylation levels of all samples were quantifiable. Therefore, the correlation between *TSLP* methylation levels, phthalate exposure, and allergic symptoms would not be biased even if *TSLP* methylation levels were decreased.

## Conclusions

Our results suggested that the higher BBzP exposure could decrease *TSLP* methylation levels, thereby increasing the risk of morning respiratory symptoms. Our findings further the understanding of the etiology of phthalate-related early biologic effects and may guide new strategies for early prevention or treatment of childhood phthalate exposure.

## Methods

### Participants

The recruited participants were children (3–12 years old) of our follow-up study, the Dampness in Buildings and Health (DBH) study. All participants lived in Tainan City, Taiwan. The DBH study investigated the correlation between indoor environment and allergies in Southern Taiwan [12]. Briefly, for analysis, we randomly selected 201 kindergartens and 259 daycare centers from 2005 to 2006. The questionnaires for assessing the children’s allergy symptoms and diseases were sent to participants by 335 successfully contacted schools. We further invited the parents of the participants to collect the environmental data related to their homes in 2007–2008. Detailed information on participant recruitment can be found in the study of [12]. Clinical diagnosis by pediatricians and environmental investigation of homes was conducted in 90 children. We used the equation below to calculate the power of our study.

$$n = \frac{{2\alpha^{2} \left( {Z_{\beta } + Z_{{\frac{\alpha }{2}}} } \right)^{2} }}{{\left( {{\text{difference}}} \right)^{2} }}$$

where *n* is sample size; difference is difference from case and control; ${\alpha }^{2}$ is standard deviation; $\beta$ is power; $\frac{\alpha }{2}$ is 0.025. In our study, sample size is 90. The difference and standard deviation are referred to the study of Wang [49] that explored the association among environmental exposure, *TSLP* methylation and allergic disease. The difference of methylation level between allergic and non-allergic disease was 5.31, and the standard deviation was 8.77. Based on such information, the power of our study was 82%, which is greater than the standard of power (80%) for adequacy in most researches. This convention represents that the probability of the type II error and the type I error was 20% and 5%, respectively. The Institutional Review Board of National Cheng Kung University Hospital approved this study (IRB NO: A-ER-105-375).

### Phthalate measurement in settled dust and urine

Dust samples were collected from beds in the major and secondary activity rooms of children using hand-held vacuum cleaners (SC-608H, SANYO Electric, Taipei, Taiwan, or VC-SP550GN, Toshiba Electronics, Taipei, Taiwan). We used a special aluminum nozzle to prevent contact with phthalate esters and collected dust in 28 × 100-mm cellulose filters (Thimble Filters Grade 84; ADVANTEC, Tokyo Roshi Kaisha, Tokyo, Japan). Dust samples were sieved using a sterile 300-μm mesh screen to remove large particles and hairs. DMP, DEP, DBP, BBzP, and DEHP levels in settled dusts were measured using GC/MS with column HP 1C (25 m $\times$ 0.2 mm, Agilent, Folsom, CA, USA). The method was modified from previous research [10]. Briefly, Fifty microgram dust samples were extracted with dichloromethane and shook ultrasonically for 30 min and the extracts were dried with nitrogen gas and reconstitution with dichloromethane. The detection limits of DMP, DEP, DBP, BBzP, and DEHP were 0.24, 0.16, 0.24, 0.15, and 0.26 mg/kg, respectively.

The first spot urine of the child on the morning of the date of the home investigation was collected by parents and stored in the freezer before our visit. Our urine containers were brown-glass and Teflon-lid bottles and were washed with methanol, hexane, and acetone. Urine samples were stored in ice and transported to the laboratory. We then separated the supernatant into different containers to analyze creatinine and phthalate metabolites. The urinary samples were extracted with acidic buffer by solid-phase extraction (SPE). Before extraction, the urinary sample were incubated with ammonium acetate and *β* -glucuronidase for 90 min. The levels of monomethyl phthalate (MMP), MEP, MBP, MBzP, and mono-2-ethylhexyl phthalate (MEHP), MEHHP, and MEOHP were measured by HPLC/MS/MS with column Mightysil RP-18 GP (L) (100 mm $\times$ 2.0 mm, 5 μm particle, Kanto Chemical Industries) and Mightysil RP-18 GP (5 mm $\times$ 2.0 mm, 5 μm particle, Kanto Chemical Industries) [96, 97].

The settled dust and urine samples were stored at − 20 °C until analysis. Detailed information on sample collection and analysis can be found in the study [12].

### Clinical and blood sample collection

The children were brought by their parents to our medical center for clinical examinations to confirm their parents’ reported health status. Pediatricians performed a physical examination. The standardized questionnaire was administered by pediatricians to examine, in detail, the severity of health outcomes, which were scored according to the reported frequency, type, areas, and size of observed symptoms. In addition, parents of participants documented the daily severity of respiratory symptoms over the past 7 days.

Blood samples were collected in vacutainers with spray-dried K2EDTA (purpletop) by the hospital staff at the time of clinical examination. Whole blood was separated into serum and buffy coat through centrifugation and stored at − 80 °C until analysis. Serum samples were used to examine the total IgE level and TSLP protein levels. Details on sample collection and analysis can be found in the study of [12].

### Cytokine analysis

Eight T_H_1-, T_H_2-, and inflammatory-related cytokines, including IL-4, IL-5, IL-6, IL-8, IL-12, IL-13, IFN-r, and TNF-α, were measured using flow cytometry. We used the BD Cytometric Bead Array Human Soluble Protein Master Buffer Kit (Biosciences, San Diego, CA, USA), which offered a broad dynamic range of fluorescence detection to quantify multiple proteins simultaneously. The detection limits of IL-4, IL-5, IL-6, IL-8, IL-12, IL-13, IFN-r, and TNF-α were 1.4, 1.1, 1.6, 1.2, 0.6, 0.6, 1.8, and 0.7 pg/mL, respectively. If concentrations of cytokines were lower than the detection limit, then half values of the lower detection limit were used. The samples were analyzed as per the instructions in the Human Soluble Protein Master Buffer Kit Instruction Manual.

### DNA and RNA extraction

DNA was isolated and purified from the buffy coats with the Quick-DNA Universal Kit (Zymo Research, Irvine, CA, USA; Cat #D4069) according to the manufacturer’s protocols. In short, 100 μL of biofluid and cell buffer and 10 μL of proteinase K were added to 100 μL of the sample. We then followed the steps mentioned in the Quick-DNA Universal Kit Manual. Finally, the DNA elution buffer was added, and the sample was centrifuged for 1 min at top speed to elute DNA.

Total RNA was extracted using NucleoZOL (Macherey‐Nagel, Düren, Germany), which was modified from the traditional method. The protocol was performed according to the NucleoZOL RNA Isolation User Manual. Briefly, we added 400 μL of NucleoZOL and 160 μL of sterile water mix to 100 μL of the sample. We then followed the steps specified in the NucleoZOL RNA Isolation User Manual. Finally, 10 μL of sterile water was added to dissolve the pellets. The value was calculate to the percent of reference/housekeeping genes (Actin Beta (ACTB)) by 2^−Δct^.

NanoDrop 2000 was used to measure DNA and RNA quality to ensure that it is adequate for reverse transcription. Reverse transcription was conducted using the GScript First-Strand Synthesis Kit (GeneDirex,, USA; Cat #MB 305-0050) and analyzed with a standard SYBR Green PCR protocol (StepOne™ Real-Time PCR System, Applied Biosystems, Carlsbad, CA, USA) to determine the gene expression level.

### Sodium bisulfite conversion and pyrosequencing assay

To quantify cytosine methylation in individual CpG sites of the candidate methylation probes identified using the methylation array, the bisulfite conversion of DNA was treated with the EpiTect Fast DNA Bisulfite Kit (QIAGEN, Germany, Cat # 59824). This treatment converts cytosine residues, but not 5-methylcytosine residues, to uracil. The uracil was transferred to thymidine during the subsequent PCR step. The sequence of *TSLP* was gained from UCSC Genome Browser on Human Dec. 2013 (GRCh38/hg38). According to the previous study, 8 CpG islands and 11 CpG sites on *TSLP* promoter in the upstream 2000 bp from the transcription start site of *TSLP* [81, 98] were chosen to design our primers using PyroMark Assay Design 2.0 (QIAGEN, Valencia, CA, USA). However, to consider the SNP effect, 33 CpG sites (25 on the CpG islands) that were published SNP sites in the promoter region were not used for our primer design. After performing PCR amplification using such primers and gel electrophoresis, only 1 CpG island and 1 CpG site (775 bp upstream from the transcription start site of *TSLP*) were amplified successfully. Briefly, bisulfite-treated DNA was amplified using the forward primer 5′- GTT TTT GGG AAG TTT TTA GGA GT-3′, biotinylated reverse primer 5′-biotin-ACT CTA ACT CCA ATT TAT CCC CTA CT-3′, and pyrosequencing sequencing primer 5′-GTG TGA GTT TTA GTA AAT GTT ATA-3′. Hot-start PCR was performed using the PyroMark PCR Kit (QIAGEN; Cat #978703). The PCR products were examined through gel electrophoresis. The biotin binding primers were combined with the streptavidin-coated beads to separate the PCR products into single strands. PyroMark Gold Q24 Reagents (QIAGEN, Cat #970802), which contained Enzyme Mixture, Substrate Mixture, dATPαS, dGTP, dCTP, and dTTP, were added into the QIAGEN cartridge. The target CpG sites were evaluated by converting the resulting programs to numerical values for peak heights. The percentage of methylation levels were analyzed with the PyroMark Q24 instrument (QIAGEN).

### Statistical analysis

Before statistical analyses, the concentration of phthalate parent compounds and metabolites were natural log-transformed and we also excluded the sample that their phthalate concentrations are below the detection limit to reduce the confounding effects of ND data. Besides, only the phthalates having > 50% of positive detection rate were further analyzed for the relationship between *TSLP* methylation levels and the phthalate exposure. Spearman’s correlation coefficients were calculated to assess the correlation between phthalate concentration and the percentage of DNA methylation. The phthalates in settled dust and urine that were significantly correlated with DNA methylation levels were analyzed using a generalized linear regression model with adjustments for child’s sex, age and BMI; parents’ smoking status, allergic history, and education levels; concentrations of PM_2.5_ and formaldehyde; temperature; and relative humidity. To understand the influence of DNA methylation levels on allergic symptoms, the Mann–Whitney U test was first performed to assess the DNA methylation percentage between the symptom and no-symptom groups. Logistic regression analyses were then used to estimate the odds ratios and 95% confidence intervals of DNA methylation levels with respiratory symptoms after adjusting for the aforementioned confounders. All tests were two-sided with a 0.05 level of significance. All statistical analyses were performed using SPSS v17 (IBM, Armonk, NY, USA).

## Supplementary Information

**Additional file 1:** The additional file showed the functionality of the specific methylation site in the control of TSLP expression.

## Data Availability

The datasets generated and/or analysed during the current study are not publicly available due [REASON WHY DATA ARE NOT PUBLIC] but are available from the corresponding author on reasonable request.

## Abbreviations

95% CI: 95% Confidence interval
*β*: Regression coefficient
BBzP: Benzyl butyl phthalate
CpG: Cytosine phosphate Guanine
DBH: Dampness in buildings and health study
DBP: Dibutyl phthalate
DEP: Diethyl phthalate
DEHP: Di-2-ethylhexyl phthalate
DMP: Dimethyl phthalate
FITC: Fluorescein isothiocyanate
ILC2s: Group 2 innate lymphoid cells
IL-4: Interleukin-4
IL-5: Interleukin-5
IL-6: Interleukin-6
IL-8: Interleukin-8
IL-13: Interleukin-13
IL-25: Interleukin-25
IL-33: Interleukin-33
LEP: Leptin
IL-4R: Interleukin-4 receptor
MBP: Mono-butyl phthalate
MBzP: Mono-benzyl phthalate
MEP: Monoethyl phthalate
MEHP: Mono-(2-ethylhexyl) phthalate
MEHHP: Mono(2-ethyl-5-hydroxyhexyl) phthalate
MEOHP: Mono-(2-ethyl-5-oxohexyl) phthalate
MiBP: Mono-isobutyl phthalate
MMP: Mono-methyl phthalate
mRNA: Messenger RNA
*N*: Sample size
RfD: Reference dose
TNF-*α*: Tumor necrosis factor-*α*
TSLP: Thymic stromal lymphopoietin
PAEs: Phthalate esters
SNP: Single nucleotide polymorphism

## References

[1] Hopp RJ, Bewtra AK, Watt GD, Nair NM, Townley RG (1984). Genetic analysis of allergic disease in twins. J Allergy Clin Immunol.

[2] Holloway JW, Yang IA, Holgate ST (2010). Genetics of allergic disease. J Allergy Clin Immunol.

[3] Portelli MA, Hodge E, Sayers I (2015). Genetic risk factors for the development of allergic disease identified by genome-wide association. Clin Exp Allergy.

[4] SoRelle J, Chen Z, Wang J, Tang M, Li X, Beutler B. Forward genetic screen of allergy reveals genetic basis for allergic disease. Am Assoc Immnol; 2018.

[5] Lu C, Hu X, Miao Y, Jiang W, Xiang Y, Deng Q (2018). Early life exposure to environmental pollution increases childhood asthma, allergy and infection. Chin Sci Bull.

[6] Lu C, Tsai T, Su P, Sun C, Wen H, Wang C, Wang S (2019). Prenatal exposure to phthalate esters and its associations with childhood allergies (TMICS Study). Environ Epidemiol.

[7] Kimber I, Pieters R (2013). Household chemicals, immune function, and allergy: a commentary. J Immunotoxicol.

[8] Araki A, Bamai YA, Bastiaensen M, Van den Eede N, Kawai T, Tsuboi T, Miyashita C, Itoh S, Goudarzi H, Konno S (2020). Combined exposure to phthalate esters and phosphate flame retardants and plasticizers and their associations with wheeze and allergy symptoms among school children. Environ Res.

[9] Mendell MJ (2007). Indoor residential chemical emissions as risk factors for respiratory and allergic effects in children: a review. Indoor Air.

[10] Bornehag C-G, Sundell J, Weschler CJ, Sigsgaard T, Lundgren B, Hasselgren M, Hägerhed-Engman L (2004). The association between asthma and allergic symptoms in children and phthalates in house dust: a nested case–control study. Environ Health Perspect.

[11] Chang H-W (2011). Phthalate esters exposure and profiles of allergic and inflammatory related cytokines in children.

[12] Hsu NY, Lee CC, Wang JY, Li YC, Chang HW, Chen CY, Bornehag CG, Wu PC, Sundell J, Su HJ (2012). Predicted risk of childhood allergy, asthma, and reported symptoms using measured phthalate exposure in dust and urine. Indoor Air.

[13] Bekö G, Weschler CJ, Langer S, Callesen M, Toftum J, Clausen G (2013). Children’s phthalate intakes and resultant cumulative exposures estimated from urine compared with estimates from dust ingestion, inhalation and dermal absorption in their homes and daycare centers. PLoS ONE.

[14] van den Driesche S, Kolovos P, Platts S, Drake AJ, Sharpe RM (2012). Inter-relationship between testicular dysgenesis and Leydig cell function in the masculinization programming window in the rat. PLoS ONE.

[15] Koch HM, Lorber M, Christensen KL, Palmke C, Koslitz S, Bruning T (2013). Identifying sources of phthalate exposure with human biomonitoring: results of a 48h fasting study with urine collection and personal activity patterns. Int J Hyg Environ Health.

[16] Markit I: Chemical economics handbook. Retrieved February 2016, 6:2017.

[17] CEH: Plasticizers. In: IHS Markit.

[18] Pei X, Song M, Guo M, Mo F, Shen X (2013). Concentration and risk assessment of phthalates present in indoor air from newly decorated apartments. Atmos Environ.

[19] Tran TM, Kannan K (2015). Occurrence of phthalate diesters in particulate and vapor phases in indoor air and implications for human exposure in Albany, New York, USA. Arch Environ Contam Toxicol.

[20] Kanazawa A, Saito I, Araki A, Takeda M, Ma M, Saijo Y, Kishi R (2010). Association between indoor exposure to semi-volatile organic compounds and building-related symptoms among the occupants of residential dwellings. Indoor Air.

[21] Xu Y, Cohen Hubal EA, Little JC (2009). Predicting residential exposure to phthalate plasticizer emitted from vinyl flooring: sensitivity, uncertainty, and implications for biomonitoring. Environ Health Perspect.

[22] Koch HM, Rossbach B, Drexler H, Angerer J (2003). Internal exposure of the general population to DEHP and other phthalates–determination of secondary and primary phthalate monoester metabolites in urine. Environ Res.

[23] Becker K, Seiwert M, Angerer J, Heger W, Koch HM, Nagorka R, Roßkamp E, Schlüter C, Seifert B, Ullrich D (2004). DEHP metabolites in urine of children and DEHP in house dust. Int J Hyg Environ Health.

[24] Koch HM, Preuss R, Angerer J: Di(2-ethylhexyl)phthalate (DEHP): human metabolism and internal exposure-- an update and latest results. Int J Androl. 2006; 29(1):155–165; discussion 181–155. 10.1111/j.1365-2605.2005.00607.x 16466535

[25] Wormuth M, Scheringer M, Vollenweider M, Hungerbühler K (2006). What are the sources of exposure to eight frequently used phthalic acid esters in Europeans?. Risk Anal Off Publ Soc Risk Anal.

[26] Huang P-C, Tsai C-H, Chen C-C, Wu M-T, Chen M-L, Wang S-L, Chen B-H, Lee C-C, Jaakkola JJ, Wu W-C (2017). Intellectual evaluation of children exposed to phthalate-tainted products after the 2011 Taiwan phthalate episode. Environ Res.

[27] Chen C-Y, Chou Y-Y, Lin S-J, Lee C-C (2015). Developing an intervention strategy to reduce phthalate exposure in Taiwanese girls. Sci Total Environ.

[28] Frederiksen H, Aksglaede L, Sorensen K, Skakkebaek NE, Juul A, Andersson A-M (2011). Urinary excretion of phthalate metabolites in 129 healthy Danish children and adolescents: estimation of daily phthalate intake. Environ Res.

[29] Frederiksen H, Nielsen JKS, Mørck TA, Hansen PW, Jensen JF, Nielsen O, Andersson A-M, Knudsen LE (2013). Urinary excretion of phthalate metabolites, phenols and parabens in rural and urban Danish mother–child pairs. Int J Hyg Environ Health.

[30] Suzuki Y, Yoshinaga J, Mizumoto Y, Serizawa S, Shiraishi H (2012). Foetal exposure to phthalate esters and anogenital distance in male newborns. Int J Androl.

[31] Kobrosly RW, Evans S, Miodovnik A, Barrett ES, Thurston SW, Calafat AM, Swan SH (2014). Prenatal phthalate exposures and neurobehavioral development scores in boys and girls at 6–10 years of age. Environ Health Perspect.

[32] Polanska K, Ligocka D, Sobala W, Hanke W (2014). Phthalate exposure and child development: the Polish Mother and Child Cohort Study. Early Human Dev.

[33] Ku H-Y, Tsai T-L, Wang P-L, Su P-H, Sun C-W, Wang C-J, Wang S-L (2020). Prenatal and childhood phthalate exposure and attention deficit hyperactivity disorder traits in child temperament: a 12-year follow-up birth cohort study. Sci Total Environ.

[34] Muerköster A-P, Frederiksen H, Juul A, Andersson A-M, Jensen RC, Glintborg D, Kyhl HB, Andersen MS, Timmermann CAG, Jensen TK (2020). Maternal phthalate exposure associated with decreased testosterone/LH ratio in male offspring during mini-puberty. Odense Child Cohort. Environ Int.

[35] Kolarik B, Naydenov K, Larsson M, Bornehag C-G, Sundell J (2008). The association between phthalates in dust and allergic diseases among Bulgarian children. Environ Health Perspect.

[36] Bamai YA, Shibata E, Saito I, Araki A, Kanazawa A, Morimoto K, Nakayama K, Tanaka M, Takigawa T, Yoshimura T (2014). Exposure to house dust phthalates in relation to asthma and allergies in both children and adults. Sci Total Environ.

[37] Larsen ST, Hansen JS, Thygesen P, Begtrup M, Poulsen OM, Nielsen GDJT. Adjuvant and immuno-suppressive effect of six monophthalates in a subcutaneous injection model with BALB/c mice. 2001; 169(1):37–51. 10.1016/s0300-483x(01)00484-x 11696408

[38] Butala JH, David RM, Gans G, McKee RH, Guo TL, Peachee VL, White Jr KLJT. Phthalate treatment does not influence levels of IgE or Th2 cytokines in B6C3F1 mice. 2004; 201(1–3):77–85. 10.1016/j.tox.2004.04.004 15297022

[39] Larsen ST, Hansen JS, Hansen EW, Clausen PA, Nielsen GD (2007). Airway inflammation and adjuvant effect after repeated airborne exposures to di-(2-ethylhexyl) phthalate and ovalbumin in BALB/c mice. Toxicology.

[40] Deutschle T, Reiter R, Butte W, Heinzow B, Keck T, Riechelmann HJE. A controlled challenge study on di (2-ethylhexyl) phthalate (DEHP) in house dust and the immune response in human nasal mucosa of allergic subjects. 2008; 116(11):1487–1493. 10.1289/ehp.11474 PMC2592268 19057701

[41] Guo J, Han B, Qin L, Li B, You H, Yang J, Liu D, Wei C, Nanberg E, Bornehag C-GJPo. Pulmonary toxicity and adjuvant effect of di-(2-exylhexyl) phthalate in ovalbumin-immunized BALB/c mice. 2012; 7(6):e39008. 10.1371/journal.pone.0039008 PMC3373502 22701742

[42] Nishioka J, Iwahara C, Kawasaki M, Yoshizaki F, Nakayama H, Takamori K, Ogawa H, Iwabuchi KJIR. Di-(2-ethylhexyl) phthalate induces production of inflammatory molecules in human macrophages. 2012; 61(1):69–78. 10.1007/s00011-011-0390-x 22005928

[43] Wang I-J, Karmaus WJ (2017). Oxidative stress-related genetic variants may modify associations of phthalate exposures with asthma. Int J Environ Res Public Health.

[44] Handy DE, Castro R, Loscalzo J (2011). Epigenetic modifications: basic mechanisms and role in cardiovascular disease. Circulation.

[45] Holbrook JD (2015). An epigenetic escape route. Trends Genet.

[46] Martin EM, Fry RC (2018). Environmental influences on the epigenome: exposure-associated DNA methylation in human populations. Annu Rev Public Health.

[47] Ramchandani S, Bhattacharya SK, Cervoni N, Szyf M (1999). DNA methylation is a reversible biological signal. Proc Natl Acad Sci.

[48] Herb BR, Wolschin F, Hansen KD, Aryee MJ, Langmead B, Irizarry R, Amdam GV, Feinberg AP (2012). Reversible switching between epigenetic states in honeybee behavioral subcastes. Nat Neurosci.

[49] Wang I, Chen S, Lu T, Chuang E, Chen P (2013). Prenatal smoke exposure, DNA methylation, and childhood atopic dermatitis. Clin Exp Allergy.

[50] Wang IJ, Karmaus WJJ (2015). The effect of phthalate exposure and filaggrin gene variants on atopic dermatitis. Environ Res.

[51] Wang I-J, Karmaus WJ, Chen S-L, Holloway JW, Ewart S (2015). Effects of phthalate exposure on asthma may be mediated through alterations in DNA methylation. Clin Epigenet.

[52] Jahreis S, Trump S, Bauer M, Bauer T, Thürmann L, Feltens R, Wang Q, Gu L, Grützmann K, Röder S (2018). Maternal phthalate exposure promotes allergic airway inflammation over 2 generations through epigenetic modifications. J Allergy Clin Immunol.

[53] Soto-Ramírez N, Arshad SH, Holloway JW, Zhang H, Schauberger E, Ewart S, Patil V, Karmaus W (2013). The interaction of genetic variants and DNA methylation of the interleukin-4 receptor gene increase the risk of asthma at age 18 years. Clin Epigenet.

[54] Patil VK, Holloway JW, Zhang H, Soto-Ramirez N, Ewart S, Arshad SH, Karmaus W (2013). Interaction of prenatal maternal smoking, interleukin 13 genetic variants and DNA methylation influencing airflow and airway reactivity. Clin Epigenet.

[55] Lockett GA, Soto-Ramírez N, Ray MA, Everson TM, Xu CJ, Patil VK, Terry W, Kaushal A, Rezwan FI, Ewart SL (2016). Association of season of birth with DNA methylation and allergic disease. Allergy.

[56] Arshad SH, Holloway JW, Karmaus W, Zhang H, Ewart S, Mansfield L, Matthews S, Hodgekiss C, Roberts G, Kurukulaaratchy R (2018). Cohort profile: the Isle of Wight whole population birth cohort (IOWBC). Int J Epidemiol.

[57] Singh S, Li SS (2012). Epigenetic effects of environmental chemicals bisphenol A and phthalates. Int J Mol Sci.

[58] Kamstra JH, Sales LB, Alestrom P, Legler J (2017). Differential DNA methylation at conserved non-genic elements and evidence for transgenerational inheritance following developmental exposure to mono(2-ethylhexyl) phthalate and 5-azacytidine in zebrafish. Epigenet Chromatin.

[59] Wu H, Estill MS, Shershebnev A, Suvorov A, Krawetz SA, Whitcomb BW, Dinnie H, Rahil T, Sites CK, Pilsner JR (2017). Preconception urinary phthalate concentrations and sperm DNA methylation profiles among men undergoing IVF treatment: a cross-sectional study. Human Reprod (Oxford, England).

[60] Chen CH, Jiang SS, Chang IS, Wen HJ, Sun CW, Wang SL (2018). Association between fetal exposure to phthalate endocrine disruptor and genome-wide DNA methylation at birth. Environ Res.

[61] Bowman A, Peterson KE, Dolinoy DC, Meeker JD, Sanchez BN, Mercado-Garcia A, Tellez-Rojo MM, Goodrich JM (2019). Phthalate exposures, DNA methylation and adiposity in Mexican children through adolescence. Front Public Health.

[62] Huang LL, Zhou B, Ai SH, Yang P, Chen YJ, Liu C, Deng YL, Lu Q, Miao XP, Lu WQ (2018). Prenatal phthalate exposure, birth outcomes and DNA methylation of Alu and LINE-1 repetitive elements: a pilot study in China. Chemosphere.

[63] Pen JJ, Aerts JL, Liechtenstein T, Escors D, Breckpot K. Manipulating immune regulatory pathways to enhance T cell stimulation. In: Immune response activation. IntechOpen; 2014.

[64] Fujita H, Soyka MB, Akdis M, Akdis CA (2012). Mechanisms of allergen-specific immunotherapy. Clin Transl Allergy.

[65] Larché M, Akdis CA, Valenta R (2006). Immunological mechanisms of allergen-specific immunotherapy. Nat Rev Immunol.

[66] Kidd P (2003). Th1/Th2 balance: the hypothesis, its limitations, and implications for health and disease. Altern Med Rev.

[67] Liu Y-J (2006). Thymic stromal lymphopoietin: master switch for allergic inflammation. J Exp Med.

[68] Wang Y-H, Angkasekwinai P, Lu N, Voo KS, Arima K, Hanabuchi S, Hippe A, Corrigan CJ, Dong C, Homey B (2007). IL-25 augments type 2 immune responses by enhancing the expansion and functions of TSLP-DC–activated Th2 memory cells. J Exp Med.

[69] Larson RP, Zimmerli SC, Comeau MR, Itano A, Omori M, Iseki M, Hauser C, Ziegler SF (2010). Dibutyl phthalate-induced thymic stromal lymphopoietin is required for Th2 contact hypersensitivity responses. J Immunol.

[70] Ait Bamai Y, Araki A, Kawai T, Tsuboi T, Saito I, Yoshioka E, Cong S, Kishi R (2016). Exposure to phthalates in house dust and associated allergies in children aged 6–12years. Environ Int.

[71] Bekö G, Callesen M, Weschler CJ, Toftum J, Langer S, Sigsgaard T, Høst A, Jensen TK, Clausen G (2015). Phthalate exposure through different pathways and allergic sensitization in preschool children with asthma, allergic rhinoconjunctivitis and atopic dermatitis. Environ Res.

[72] Langer S, Bekö G, Weschler CJ, Brive LM, Toftum J, Callesen M, Clausen G (2014). Phthalate metabolites in urine samples from Danish children and correlations with phthalates in dust samples from their homes and daycare centers. Int J Hyg Environ Health.

[73] Ziegler SF (2012). Thymic stromal lymphopoietin and allergic disease. J Allergy Clin Immunol.

[74] Soumelis V, Reche PA, Kanzler H, Yuan W, Edward G, Homey B, Gilliet M, Ho S, Antonenko S, Lauerma A (2002). Human epithelial cells trigger dendritic cell mediated allergic inflammation by producing TSLP. Nat Immunol.

[75] Ying S, O'Connor B, Ratoff J, Meng Q, Mallett K, Cousins D, Robinson D, Zhang G, Zhao J, Lee TH (2005). Thymic stromal lymphopoietin expression is increased in asthmatic airways and correlates with expression of Th2-attracting chemokines and disease severity. J Immunol (Baltimore, Md: 1950).

[76] Mou Z, Xia J, Tan Y, Wang X, Zhang Y, Zhou B, Li H, Han D (2009). Overexpression of thymic stromal lymphopoietin in allergic rhinitis. Acta Otolaryngol.

[77] Podlecka D, Gromadzińska J, Mikołajewska K, Fijałkowska B, Stelmach I, Jerzynska J (2020). Longitudinal effect of phthalates exposure on allergic diseases in children. Ann Allergy Asthma Immunol.

[78] Callesen M, Bekö G, Weschler CJ, Langer S, Brive L, Clausen G, Toftum J, Sigsgaard T, Høst A, Jensen TK (2014). Phthalate metabolites in urine and asthma, allergic rhinoconjunctivitis and atopic dermatitis in preschool children. Int J Hyg Environ Health.

[79] van Bodegom D, Zhong J, Kopp N, Dutta C, Kim MS, Bird L, Weigert O, Tyner J, Pandey A, Yoda A (2012). Differences in signaling through the B-cell leukemia oncoprotein CRLF2 in response to TSLP and through mutant JAK2. Blood.

[80] Cianferoni A, Spergel J (2014). The importance of TSLP in allergic disease and its role as a potential therapeutic target. Expert Rev Clin Immunol.

[81] Luo Y, Zhou B, Zhao M, Tang J, Lu Q (2014). Promoter demethylation contributes to TSLP overexpression in skin lesions of patients with atopic dermatitis. Clin Exp Dermatol.

[82] Junge KM, Bauer T, Geissler S, Hirche F, Thürmann L, Bauer M, Trump S, Bieg M, Weichenhan D, Gu L (2016). Increased vitamin D levels at birth and in early infancy increase offspring allergy risk-evidence for involvement of epigenetic mechanisms. J Allergy Clin Immunol.

[83] Henry EK, Inclan-Rico JM, Siracusa MC (2017). Type 2 cytokine responses: regulating immunity to helminth parasites and allergic inflammation. Curr Pharmacol Rep.

[84] Burks AW, Palmer KP, Haith MM, Benson JB (2008). Allergies. Encyclopedia of infant and early childhood development.

[85] Kubo T, Morita H, Sugita K, Akdis CA, O'Hehir RE, Holgate ST, Sheikh A (2017). Chapter 1 - Introduction to mechanisms of allergic diseases. Middleton's allergy essentials.

[86] Calzada D, Baos S, Cremades-Jimeno L, Cárdaba B (2018). Immunological mechanisms in allergic diseases and allergen tolerance: the role of treg cells. J Immunol Res.

[87] Palomares O, Martín-Fontecha M, Lauener R, Traidl-Hoffmann C, Cavkaytar O, Akdis M, Akdis CA (2014). Regulatory T cells and immune regulation of allergic diseases: roles of IL-10 and TGF- *β*. Genes Immun.

[88] Bernink J, Mjösberg J, Spits H (2013). Th1-and Th2-like subsets of innate lymphoid cells. Immunol Rev.

[89] Bernink JH, Germar K, Spits H (2014). The role of ILC2 in pathology of type 2 inflammatory diseases. Curr Opin Immunol.

[90] Ito T, Wang YH, Duramad O, Hori T, Delespesse GJ, Watanabe N, Qin FXF, Yao Z, Cao W, Liu YJ (2005). TSLP-activated dendritic cells induce an inflammatory T helper type 2 cell response through OX40 ligand. J Exp Med.

[91] Licona-Limón P, Kim LK, Palm NW, Flavell RA (2013). T H 2, allergy and group 2 innate lymphoid cells. Nat Immunol.

[92] Wang Q, Du J, Zhu J, Yang X, Zhou B (2015). Thymic stromal lymphopoietin signaling in CD4+ T cells is required for TH2 memory. J Allergy Clin Immunol.

[93] Stier MT, Bloodworth MH, Toki S, Newcomb DC, Goleniewska K, Boyd KL, Quitalig M, Hotard AL, Moore ML, Hartert TV (2016). Respiratory syncytial virus infection activates IL-13–producing group 2 innate lymphoid cells through thymic stromal lymphopoietin. J Allergy Clin Immunol.

[94] Shabihkhani M, Lucey GM, Wei B, Mareninov S, Lou JJ, Vinters HV, Singer EJ, Cloughesy TF, Yong WH (2014). The procurement, storage, and quality assurance of frozen blood and tissue biospecimens in pathology, biorepository, and biobank settings. Clin Biochem.

[95] Slieker RC, Relton CL, Gaunt TR, Slagboom PE, Heijmans BT (2018). Age-related DNA methylation changes are tissue-specific with ELOVL2 promoter methylation as exception. Epigenet Chromatin.

[96] Silva MJ, Barr DB, Reidy JA, Malek NA, Hodge CC, Caudill SP, Brock JW, Needham LL, Calafat AM (2004). Urinary levels of seven phthalate metabolites in the U.S. population from the National Health and Nutrition Examination Survey (NHANES) 1999–2000. Environ Health Perspect.

[97] Inoue K, Higuchi T, Okada F, Iguchi H, Yoshimura Y, Sato A, Nakazawa H (2003). The validation of column-switching LC/MS as a high-throughput approach for direct analysis of di (2-ethylhexyl) phthalate released from PVC medical devices in intravenous solution. J Pharm Biomed Anal.

[98] Luis FM, Andrea MR, Juan BG, Luz H, Diana A, Cesar M, Beatriz M, Javier MC. Methylation patterns near and inside of the TSLP promoter in an African descendent population of Colombia. 2013.

