# Supplementary material for: Associations among phthalate exposure, DNA methylation of TSLP, and childhood allergy
Source: Clin Epigenetics. 2021 Apr 9;13:76. doi: 10.1186/s13148-021-01061-1 (PMC8035749; doi:10.1186/s13148-021-01061-1)
Supplement: Supplementary file 1 — Additional file 1: The additional file showed the functionality of the specific methylation site in the control of TSLP expression. [file 13148_2021_1061_MOESM1_ESM.docx]

**Supplementary information**


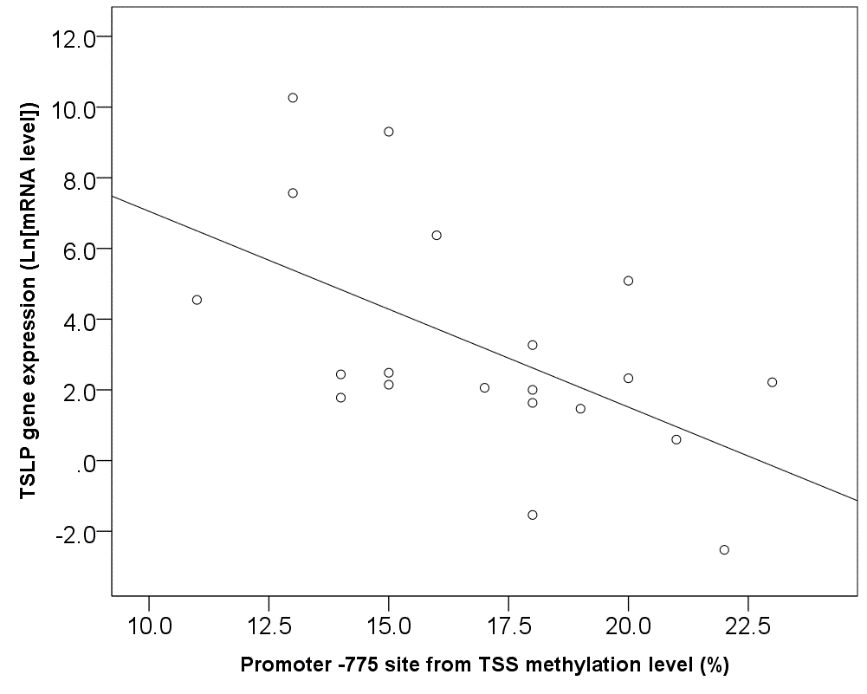


r = -0.553*

**Figure S1. The correlation between *TSLP* DNA methylation level and mRNA expression.** * *P*<0.05, by Spearman correlation coefficient

a


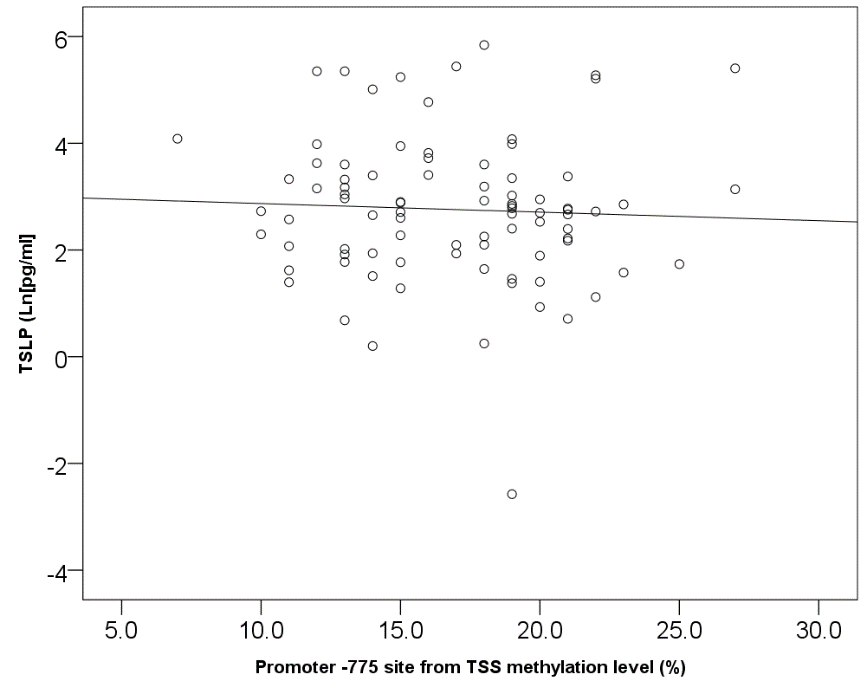


r = -0.089

b


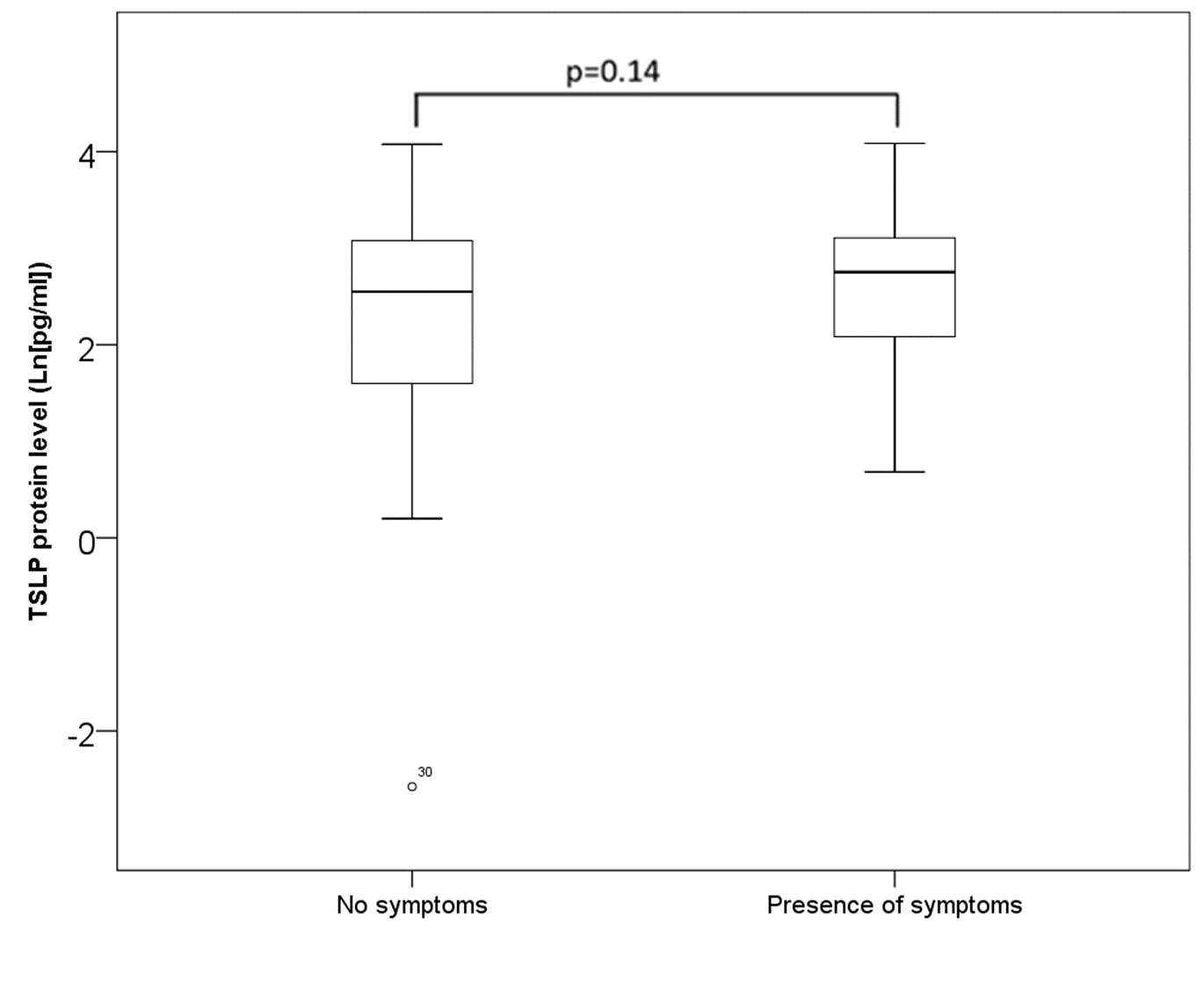


**Figure S2. (a) The correlation between *TSLP* DNA methylation level and TSLP protein concentration, (b) The difference between TSLP levels and presences of symptoms.**

* *P*<0.05, by Spearman correlation coefficient (a)

**p*<0.05, by Mann-Whitney U test (b)

a


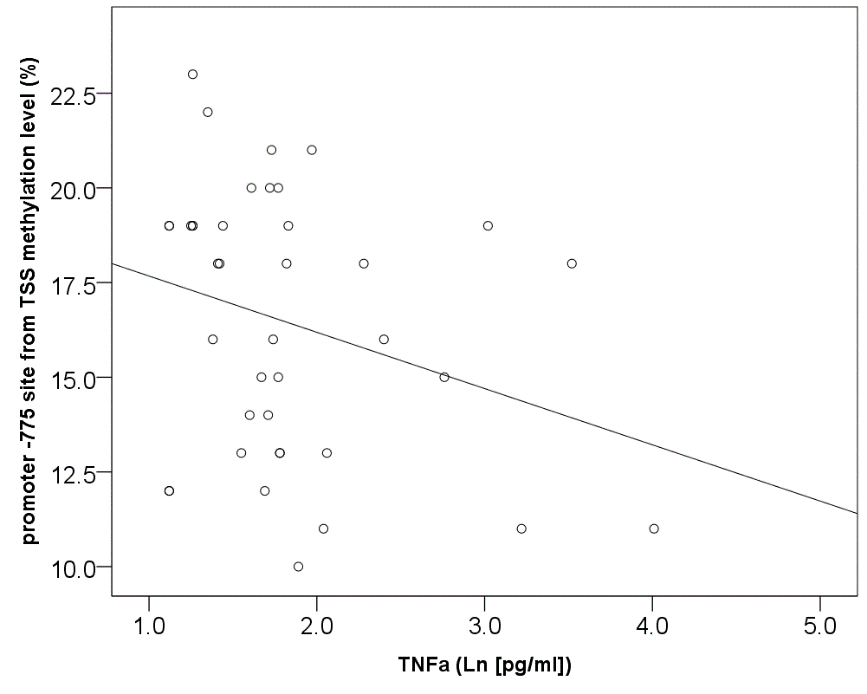


b

r = -0.287


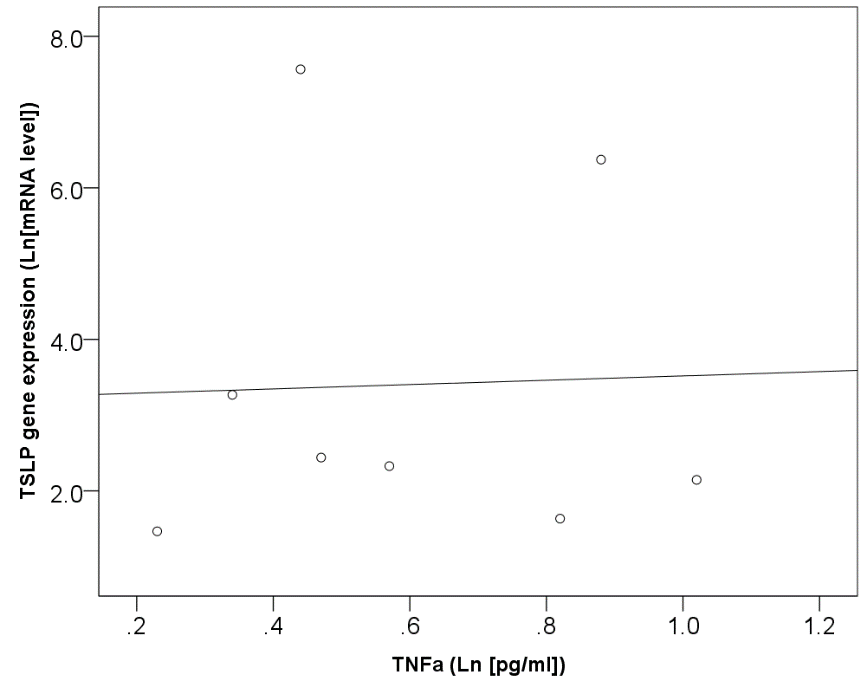


r = 0.035

**Figure S3. (a) The correlation between *TSLP* DNA methylation level and TNF-α concentration. (b) The correlation between *TSLP* mRNA expression and TNF-α concentration.**

* *P*<0.05, by Spearman correlation coefficient
